# Extended Anticoagulation Therapy With Rivaroxaban for Cancer‐Associated Low‐Risk Pulmonary Embolism According to Different Performance Status Scores: Insights From the ONCO PE Randomized Trial

**DOI:** 10.1161/JAHA.125.045541

**Published:** 2026-02-27

**Authors:** Wei Xiong, Yugo Yamashita, Takeshi Morimoto, Nao Muraoka, Wataru Shioyama, Yoshihisa Nakagawa, Ryuki Chatani, Hiromi Yamamoto, Tatsuhiro Shibata, Nagisa Morikawa, Yoshihiro Fukumoto, Yuji Nishimoto, Koh Ono, Takeshi Kimura, the ONCO PE Trial Investigators

**Affiliations:** ^1^ Department of Pulmonary and Critical Care Medicine Xinhua Hospital, Shanghai Jiaotong University School of Medicine Shanghai China; ^2^ Department of Cardiovascular Medicine Graduate School of Medicine, Kyoto University Kyoto Japan; ^3^ Department of Data Science Hyogo Medical University Nishinomiya Japan; ^4^ Division of Cardiology Shizuoka Cancer Center Shizuoka Japan; ^5^ Department of Cardiovascular Medicine Shiga University of Medical Science Otsu Shiga Japan; ^6^ Department of Cardiovascular Medicine Kurashiki Central Hospital Kurashiki Japan; ^7^ Division of Cardiovascular Medicine, Department of Internal Medicine Kurume University School of Medicine Kurume Japan; ^8^ Division of Cardiology Osaka General Medical Center Osaka Japan; ^9^ Department of Cardiology Hirakata Kohsai Hospital Hirakata Japan

**Keywords:** cancer, extended anticoagulation, performance status score, pulmonary embolism, rivaroxaban, Embolism, Thrombosis

## Abstract

**Background:**

The ONCO PE (Optimal Duration of Anticoagulation Therapy for Low‐Risk Pulmonary Embolism Patients With Cancer) trial demonstrated the superiority of 18‐month compared with 6‐month rivaroxaban treatment for cancer‐associated low‐risk pulmonary embolism in reducing recurrent venous thromboembolism. However, it was uncertain whether the results could be applicable to patients with different performance status (PS) scores, which evaluate the physical condition of patients with cancer undergoing anticancer treatment.

**Methods:**

In this post hoc subgroup analysis of the ONCO PE trial, we compared the 18‐month and 6‐month rivaroxaban treatment groups in 2 subgroups: the low PS score (no restricted physical activity: PS=0; n=79) and high PS score (restricted physical activity: PS ≥1; n=99) subgroups. The primary end point was recurrent venous thromboembolism, and the major secondary end point was major bleeding.

**Results:**

The rate of recurrent venous thromboembolism was lower in the 18‐month rivaroxaban group than in the 6‐month rivaroxaban group, significantly among the low PS score subgroup (2.7% versus 19.0%, *P*=0.049) and numerically among the high PS score subgroup without statistical significance (7.7% versus 19.1%, *P*=0.10). The rate of major bleeding was not different between the 2 groups among the low PS score subgroup (2.7% versus 7.1%, *P*=0.39), while it was numerically higher in the 18‐month rivaroxaban group than in the 6‐month rivaroxaban group among the high PS score subgroup, without statistical significance (11.5% versus 4.3%, *P*=0.20).

**Conclusions:**

Extended anticoagulation therapy for patients with cancer‐associated low‐risk pulmonary embolism might have a potential benefit in reducing thrombotic risk irrespective of PS score, whereas there might be some concerns on an increased risk of major bleeding in patients with a high PS score.

**Registration:**

URL: https://www.clinicaltrials.gov; Unique Identifier: NCT04724460.

**Nonstandard Abbreviations and Acronyms**
ECOG: Eastern Cooperative Oncology Group
ONCO PE: Optimal Duration of Anticoagulation Therapy for Low‐Risk Pulmonary Embolism Patients With Cancer
PS: performance status
sPESI: simplified Pulmonary Embolism Severity Index

**Clinical Perspective**

**What Is New?**

- This subgroup analysis of the ONCO PE (Optimal Duration of Anticoagulation Therapy for Low‐Risk Pulmonary Embolism Patients With Cancer) trial demonstrated that the benefit of 18‐month over 6‐month rivaroxaban anticoagulation treatment for reducing recurrent venous thromboembolism in cancer‐associated acute low‐risk pulmonary embolism was consistent irrespective of patients' performance status score, which is a key indicator of functional capacity and prognosis in oncology, with a numerical increase in major bleeding events among patients with high performance status (≥1) score.

**What Are the Clinical Implications?**

- These findings support the decision of extended anticoagulation with rivaroxaban for patients with cancer‐associated acute low‐risk pulmonary embolism to mitigate recurrent thrombotic risk, regardless of their performance status.
- Although extended anticoagulation for patients with cancer‐associated acute low‐risk pulmonary embolism should be a standard regimen, the decision must be individualized, with particular caution exercised for patients with a high performance status score whose bleeding risk might compromise the thromboprophylactic benefit.

Venous thromboembolism (VTE), which comprises pulmonary embolism (PE) and deep vein thrombosis, is an important disease that affects nearly 10 million people every year worldwide, and PE is one of the major causes of morbidity and mortality in patients with cancer.^1^, ^2^ The current guidelines have recommended a minimum of 6 months of anticoagulation therapy for cancer‐associated PE,^3^, ^4^, ^5^, ^6^, ^7^, ^8^ although extended anticoagulation therapy beyond 6 months has been a matter of active debate partly due to the lack of solid evidence.^9^ Recently, the ONCO PE (Optimal Duration of Anticoagulation Therapy for Low‐Risk Pulmonary Embolism Patients With Cancer) trial reported that 18‐month rivaroxaban treatment was superior to 6‐month rivaroxaban treatment with respect to recurrent VTE events in patients with cancer who have acute low‐risk PE with a simplified Pulmonary Embolism Severity Index (sPESI) score of 1.^10^ Given the dilemma of balancing thrombotic and bleeding risks with extended anticoagulation therapy, a personalized approach that takes patient characteristics into account could be helpful for the optimal implementation of extended anticoagulation therapy in an individual patient with cancer‐associated VTE.^9^

Eastern Cooperative Oncology Group (ECOG) performance status (PS) score is an essential score for the evaluation of physical condition of patients with cancer undergoing anticancer treatment, which categorizes physical activity from 0 to 4, with a higher score indicating more restricted physical activity. The PS score could be helpful for considering the indication for anticancer treatment.^11^, ^12^, ^13^ In fact, a high PS score has been reported to be independently associated with an increased risk of mortality in patients with cancer, which could be used for decision‐making of palliative care without aggressive anticancer treatment in daily clinical practice.^13^ Thus, clinicians should take PS score into consideration for the management of patients with cancer. Furthermore, a high PS score was reported to be associated with an increased risk of thrombosis.^14^, ^15^, ^16^, ^17^, ^18^ In this context, PS score could also be useful for anticoagulation management strategies, and it may be important whether the results of the ONCO PE trial could be applicable to patients with different PS scores. Therefore, we conducted a post hoc subgroup analysis of the ONCO PE trial to evaluate the impact of PS score on clinical outcomes between the 18‐month and 6‐month rivaroxaban treatment groups.

## METHODS

### Study Oversight

The data that support the findings of this study are available from the corresponding author on reasonable request. The ONCO PE trial (NCT04724460) was an investigator‐initiated, multicenter, open‐label, adjudicator‐blinded, superiority, randomized clinical trial that compared 18‐month rivaroxaban treatment with 6‐month rivaroxaban treatment in patients with cancer and acute low‐risk PE at 32 medical institutions in Japan (Data S1). Full details of the trial were previously described.^10^ The ONCO PE trial was conducted in line with the principles of the Declaration of Helsinki, and was approved by the Kyoto University institutional review board, along with the institutional review boards of all participating institutions (Data S2). All patients signed written informed consent. The current study was reported in accordance with the CONSORT (Consolidated Standards of Reporting Trials) 2025 guidelines.^19^

### Study Population

The ONCO PE trial included adult patients with active cancer and newly diagnosed acute low‐risk PE confirmed by computed tomography pulmonary angiography.

Active cancer was defined as cancer that met one of the following criteria: newly diagnosed cancer within 6 months of randomization; cancer treatment performed within 6 months of randomization; current cancer treatment; recurrence, local invasion, or distant metastases; or a hematopoietic malignancy that has not achieved complete remission.^20^ Acute low‐risk PE was defined as acute PE in patients with an sPESI score of 1 (no score components other than cancer). The full detailed inclusion and exclusion criteria are provided in Data S3.

From February 2021 to March 2023, a total of 179 patients were enrolled and randomized.

**Figure 1:**
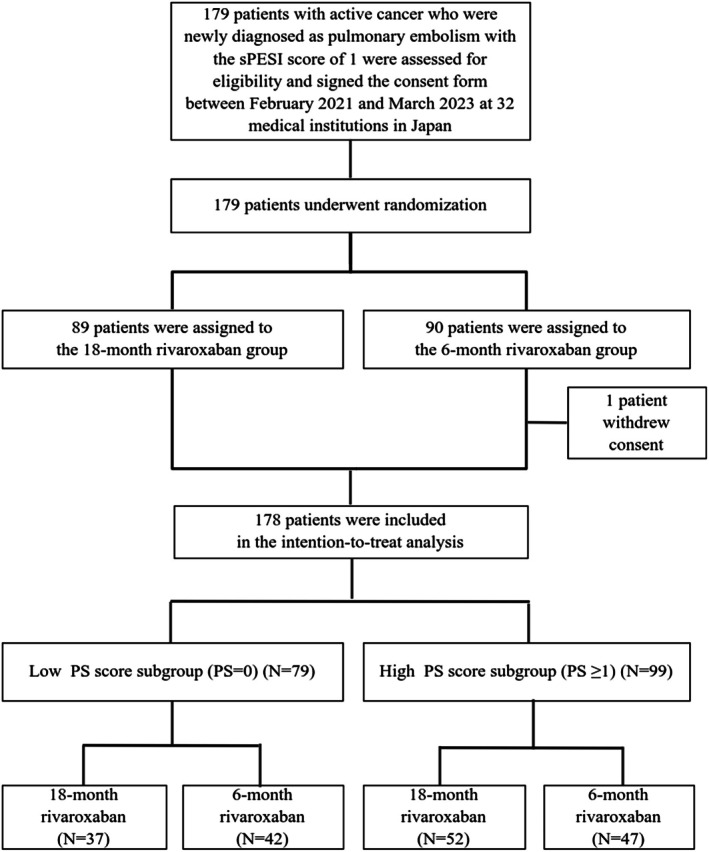
Study flowchart. ECOG PS score was defined as follows: 0: fully active at predisease performance levels without restriction; 1: restricted physically strenuous activity but ambulatory and able to perform work of a light and sedentary nature; 2: ambulatory and capable of all self‐care but unable to perform any work activities; 3: capable of only limited self‐care, and confined to bed or a chair more than 50% of waking hours; and 4: completely disabled, cannot perform any self‐care, and totally confined to bed or a chair. ECOG indicates Eastern Cooperative Oncology Group; PS, performance status; and sPESI, simplified Pulmonary Embolism Severity Index.

After excluding 1 patient who withdrew consent during follow‐up, 178 patients were included in the current study (Figure 1). Based on the previous literature,^13^ the current study population was divided into 2 subgroups: the low PS score subgroup, which was defined as patients with a PS score of 0, and the high PS score subgroup, which was defined as patients with a PS score of ≥1. ECOG PS score was defined as follows: 0: fully active at predisease performance levels without restriction; 1: restricted physically strenuous activity but ambulatory and able to perform work of a light and sedentary nature; 2: ambulatory and capable of all self‐care but unable to perform any work activities; 3: capable of only limited self‐care, and confined to bed or a chair >50% of waking hours; and 4: completely disabled, cannot perform any self‐care and totally confined to bed or a chair. Detailed definitions of patient characteristics are provided in Data S4.

### Clinical End Points

The primary and major secondary end points of the current study were identical to those used in the primary report.^10^ The members of an independent clinical events committee (Data S1) who were unaware of the assignments of the study groups adjudicated all suspected outcome events, using a prespecified criteria.^10^ The adjudication was conducted with a reference of detailed clinical course of each event, which was recorded using an electronic case report form in a web‐based database system.

The primary end point was recurrent VTE event during the 18 months after the PE diagnosis. Recurrent VTE was defined as PE and/or deep vein thrombosis, which referred to the appearance of new or worsening thromboembolism on imaging tests of computed tomography pulmonary angiography, pulmonary ventilation perfusion scintigraphy, ultrasonography of lower‐limb vein system, and venography, irrespective of PE symptoms.

The major secondary end point was a major bleeding event during the 18 months after the PE diagnosis. According to the definition of the International Society on Thrombosis and Hemostasis criteria,^21^ major bleeding was defined as fatal bleeding, symptomatic bleeding in a critical area or organ, and bleeding causing a reduction in the hemoglobin level of ≥2 g/dL or leading to a transfusion of ≥2 U of whole blood or red blood cells. Other secondary end points were all‐cause death, symptomatic recurrent VTE, and all clinically relevant bleeding events during the 18 months after the PE diagnosis. A list of the prespecified secondary end points and criteria for adjudication of all the outcomes was provided in Data S5.

### Statistical Analysis

The current study was conducted as a post hoc subgroup analysis, thus we did not conduct a power calculation due to the nature of an exploratory analysis. Continuous variables are presented either as mean and SD or as median and interquartile range, based on whether their distributions were normal. Categorical variables are presented as numbers and percentages. Continuous variables were compared using Student *t* test or Mann–Whitney *U* test, based on whether their distributions were normal. The comparison of categorical variables was conducted using the χ^2^ test or Fisher exact test. No imputation for missing data was performed. The incidence of patients with primary and secondary end points were compared between the 2 treatment groups in the intention‐to‐treat population. Odds ratios (ORs) and corresponding 95% CIs were estimated using logistic regression models. The cumulative incidences of the primary and major secondary end points were compared between the 18‐month and 6‐month rivaroxaban groups in each subgroup using the Kaplan–Meier method. The differences for the primary and major secondary end points were assessed by log‐rank test. We constructed the same logistic regression models to estimate the *P* values for any treatment‐by‐subgroup interactions. Of note, subgroup analyses were exploratory and might be underpowered, which affected the reliability of nonsignificant trends. All statistical analyses were conducted using SPSS version 27 software (IBM) and JMP version 15.2.0 (SAS Institute Inc.). Statistical significance was defined as a 2‐tailed *P* value of <0.05.

## RESULTS

### Baseline Characteristics of Patients

Among 178 patients in the current study, patients with a PS score of 0, 1, 2, and 3 accounted for 79 (44%), 82 (46%), 10 (5.6%), and 7 (3.9%), respectively. Accordingly, 79 and 99 patients were assigned to the low PS score and high PS score subgroups, respectively (Figure 1). There was no significant difference between the 2 subgroups in demographics, cancer status, comorbidities, and predisposing factors for PE, except for a higher prevalence of metastasis in the high PS score subgroup than in the low PS score subgroup (50.5% versus 21.5%, *P*<0.001) (Table 1). There was also no significant difference between the 2 subgroups in PE characteristics including thrombus location, right ventricular dysfunction, and concomitant deep vein thrombosis. The detailed cancer types are presented in Table S1. The baseline clinical characteristics were well balanced between the 18‐month and 6‐month rivaroxaban groups both in the low and high PS score subgroups (Table S2).

**Table 1:** Clinical Characteristics of Patients at Baseline

	Low PS score (PS=0) (n=79)	High PS score (PS≥1) (n=99)	*P* value
Demographics			
Age, y	65.0±10.6	66.4±10.3	0.38
Age ≥ 75 y, n (%)	16 (20.3)	24 (24.2)	0.53
Men, n (%)	36 (45.6)	47 (47.5)	0.80
Body weight, kg	61.0±12.0	59.4±10.9	0.36
Body weight ≤60 kg, n (%)	41 (51.9)	55 (55.6)	0.63
Body mass index, kg/m^2^	23.0±3.3	22.9±4.2	0.85
Cancer status, n (%)			
Newly diagnosed cancer within 6 mo	44 (55.7)	58 (58.6)	0.70
Surgery within 6 mo	34 (43.0)	35 (35.4)	0.30
Chemotherapy within 6 mo	50 (63.3)	57 (57.6)	0.44
Radiotherapy within 6 mo	5 (6.3)	8 (8.1)	0.78
Recurrent cancer	10 (12.7)	17 (17.2)	0.40
Metastasis	17 (21.5)	50 (50.5)	<0.001
Comorbidities, n (%)			
Hypertension	28 (35.4)	39 (39.4)	0.59
Diabetes	7 (8.9)	20 (20.2)	0.04
Dyslipidemia	15 (19.0)	23 (23.2)	0.49
History of stroke	0 (0.0)	1 (1.0)	1.00
History of venous thromboembolism	3 (3.8)	4 (4.0)	1.00
Autoimmune disorder	6 (7.6)	6 (6.1)	0.77
Varicose veins of lower extremities	1 (1.3)	0 (0.0)	0.44
History of major bleeding	4 (5.1)	6 (6.1)	1.00
Chronic kidney disease	7 (8.9)	5 (5.1)	0.37
Predisposing factors for PE, n (%)			
Transient factors	20 (25.3)	22 (22.2)	0.63
Immobilization	5 (6.3)	7 (7.1)	1.00
Recent surgery within 2 mo	13 (16.5)	16 (16.2)	0.96
Central venous catheterization	0 (0.0)	1 (1.0)	1.00
Onset settings, n (%)			
Out‐of‐hospital	53 (67.1)	44 (44.4)	0.003
Home treatment	40 (75.5)	26 (59.1)	0.09
In‐hospital	26 (32.9)	55 (55.6)	0.003
In‐hospital after surgery	10 (12.7)	19 (19.2)	0.24
Presentation at diagnosis			
Systolic blood pressure, mm Hg	130.5±17.6	123.4±15.6	0.005
Heart rate, beats per min	79.6±12.2	82.4±12.1	0.14
Oxygen saturation, %	97.5±1.2	96.9±1.6	0.01
Symptomatic PE, n (%)	7 (8.9)	15 (15.2)	0.25
PE characteristics, n (%)			
Thrombus location			
Central	5 (6.3)	9 (9.1)	0.78
Main	8 (10.1)	14 (14.1)	
Lobar	27 (34.2)	27 (27.3)	
Segmental	24 (30.4)	29 (29.3)	
Subsegmental	15 (19.0)	20 (20.2)	
RV dysfunction	4 (5.1)	11 (11.1)	0.18
RV/LV≥0.9	4 (5.1)	8 (8.1)	0.55
Concomitant DVT	45 (57.0)	59 (59.6)	0.72
Laboratory tests at diagnosis			
Hemoglobin, g/dL	11.8±2.2	11.1±1.9	0.03
Anemia, n (%)	45 (57.0)	73 (73.7)	0.02
Platelet count, ×100 000/μL	22.2±9.5	24.1±11.7	0.24
Platelet count <100 000/μL, n (%)	3 (3.8)	8 (8.1)	0.35
Creatinine clearance, mL/min	85.7±28.7	84.4±29.0	0.77
Creatinine clearance ≤50 mL/min, n (%)	6 (7.6)	5 (5.1)	0.54
D‐dimer (n=166), μg/mL	5.4 (2.9–11.0)	10.2 (5.2–19.1)	<0.001
NT‐proBNP (n=48), pg/mL	80.7 (53.8–187.0)	120.0 (60.2–230.3)	0.23
Troponin I (n= 53), ng/dL	0.01 (0.005–10.000)	0.01 (0.010–10.000)	0.89
Concomitant medication, n (%)			
Antiplatelet	2 (2.5)	3 (3.0)	1.00
Statins	7 (8.9)	14 (14.1)	0.35
Steroid	8 (10.1)	16 (16.2)	0.28
NSAIDs	8 (10.1)	17 (17.2)	0.20
Proton pump inhibitor	24 (30.4)	57 (57.6)	<0.001

ECOG PS score was defined as follows: 0: fully active at predisease performance levels without restriction; 1: restricted physically strenuous activity but ambulatory and able to perform work of a light and sedentary nature; 2: ambulatory and capable of all self‐care but unable to perform any work activities; 3: capable of only limited self‐ care, and confined to bed or a chair more than 50% of waking hours; 4: completely disabled, cannot perform any self‐care, and totally confined to bed or a chair. DVT indicates deep vein thrombosis; ECOG, Eastern Cooperative Oncology Group; LV, left ventricular; NT‐proBNP, N‐terminal pro‐B‐type natriuretic peptide; PE, pulmonary embolism; PS, performance status; and RV, right ventricular.

### Primary End Point

**Figure 2:**
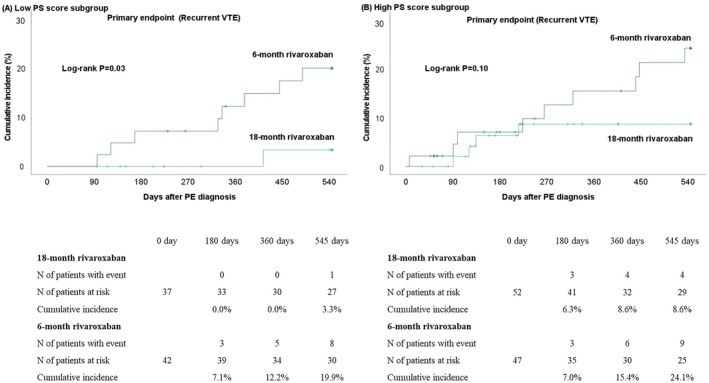
Kaplan–Meier curves of primary end point compared between 18‐month and 6‐month rivaroxaban treatment groups. **A**, Low PS score subgroup; (**B**) high PS score subgroup. The primary end point was recurrent VTE event during the 18 months after the PE diagnosis, which was defined as PE and/or DVT, which referred to the appearance of new or worsening thromboembolism on imaging tests irrespective of PE symptoms. DVT indicates deep vein thrombosis; PE, pulmonary embolism; PS, performance status; and VTE, venous thromboembolism.

In the low PS score subgroup, the primary end point of recurrent VTE occurred in 1 of 37 patients (2.7%) in the 18‐month rivaroxaban group and in 8 of 42 patients (19.0%) in the 6‐month rivaroxaban group (OR, 0.12 [95% CI, 0.01–0.99], *P*=0.049) (Table 2). In the high PS score subgroup, the primary end point of recurrent VTE occurred in 4 of 52 patients (7.7%) in the 18‐month rivaroxaban group and in 9 of 47 patients (19.1%) in the 6‐month rivaroxaban group (OR, 0.35 [95% CI, 0.10–1.23], *P*=0.10) (Table 2). There was no treatment‐by‐subgroup interaction in the effect of 18‐month relative to 6‐month rivaroxaban treatment on the recurrent VTE (*P*=0.39 for interaction). The time‐to‐event curves for recurrent VTE in the low and high PS score subgroups are provided in Figure 2A and Figure 2B, respectively.

**Table 2:** Clinical Outcomes

	No. of patients with event		Difference in the event rates (95% CI)	Odds ratio (95% CI)	*P* value	*P* for interaction
	18‐mo rivaroxaban (n=89)	6‐mo rivaroxaban (n=89)				
Primary end point (recurrent VTE)						
Low PS score (PS=0)	1/37 (2.7%)	8/42 (19.0%)	−16.3% (−29.1% to –1.5%)	0.12 (0.01–0.99)	0.049	0.39
High PS score (PS ≥1)	4/52 (7.7%)	9/47 (19.1%)	−11.5% (−24.8% to 2.5%)	0.35 (0.10–1.23)	0.10	
Major secondary end point (major bleeding)						
Low PS score (PS=0)	1/37 (2.7%)	3/42 (7.1%)	−4.4% (−14.9% to 7.0%)	0.36 (0.04–3.63)	0.39	0.15
High PS score (PS≥1)	6/52 (11.5%)	2/47 (4.3%)	7.3% (−4.4 to 18.0%)	2.94 (0.56–15.32)	0.20	

The primary end point was recurrent VTE event during the 18 months after the PE diagnosis, which was defined as PE and/or DVT, which referred to the appearance of new or worsening thromboembolism on imaging tests irrespective of PE symptoms. The major secondary end point was major bleeding event during the 18 months after the PE diagnosis. According to the definition of the International Society on Thrombosis and Hemostasis criteria, major bleeding was defined as fatal bleeding, symptomatic bleeding in a critical area or organ, and bleeding causing a reduction in the hemoglobin level of ≥2 g/dL or leading to a transfusion of ≥2 U of whole blood or red blood cells. DVT indicates deep vein thrombosis; PE, pulmonary embolism; PS, performance status; and VTE, venous thromboembolism.

### Major Secondary End Point

In the low PS score subgroup, the major secondary end point of major bleeding occurred in 1 of 37 patients (2.7%) in the 18‐month rivaroxaban group and in 3 of 42 patients (7.1%) in the 6‐month rivaroxaban group (OR, 0.36 [95% CI, 0.04–3.63], *P*=0.39) (Table 2). In the high PS score subgroup, the major secondary end point of major bleeding occurred in 6 of 52 patients (11.5%) in the 18‐month rivaroxaban group and in 2 of 47 patients (4.3%) in the 6‐month rivaroxaban group (OR, 2.94 [95% CI, 0.56–15.32], *P*=0.20) (Table 2). There was no treatment‐by‐subgroup interaction in the effect of 18‐month relative to 6‐month rivaroxaban treatment on major bleeding (*P*=0.15 for interaction).

**Figure 3:**
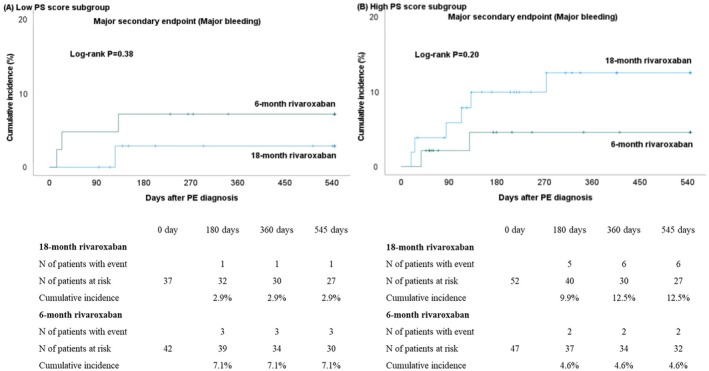
Kaplan–Meier curves of the major secondary end point compared between 18‐month and 6‐month rivaroxaban treatment groups. **A**, Low PS score subgroup; (**B**) high PS score subgroup. The major secondary end point was major bleeding event during the 18 months after the PE diagnosis. According to the definition of the International Society on Thrombosis and Hemostasis criteria, major bleeding was defined as fatal bleeding, symptomatic bleeding in a critical area or organ, and bleeding causing a reduction in the hemoglobin level of ≥2 g/dL or leading to a transfusion of ≥2 U of whole blood or red blood cells. PE indicates pulmonary embolism; and PS, performance status.

The time‐to‐event curves for major bleeding in the low and high PS score subgroups are provided in Figure 3A and Figure 3B, respectively. The detailed sites of major bleeding in the low and high PS score subgroups are also presented in Table S3.

### Other Secondary End Points

There were no statistical differences with respect to the occurrence of all‐cause death, symptomatic recurrent VTE, and all clinically relevant bleeding between the 18‐month and 6‐month rivaroxaban groups in both the low or high PS score subgroups (Table S4). The time‐to‐event curves for all‐cause death, symptomatic recurrent VTE, and all clinically relevant bleeding in the low and PS score subgroups are provided in Figure S1, Figure S2, and Figure S3, respectively. The primary and major secondary end points in each PS score are also presented in Table S5.

## DISCUSSION

The current post hoc subgroup analysis confirmed the consistent results with that of the primary analysis of the ONCO PE trial irrespective of PS scores. PS score has been used as a practical score used to guide indications for anticancer treatment and estimate prognosis in patients with cancer. In particular, high PS scores reflect poor prognosis of patients with cancer and may create therapeutic dilemmas in daily clinical practice regarding anticancer treatment and anticoagulation therapy.

It has been reported that high ECOG PS score is associated with an increased risk of development of VTE in patients with cancer.^14^, ^15^ More importantly, high ECOG PS score is associated with an increased risk of VTE recurrence in patients with cancer‐associated VTE.^16^, ^17^, ^18^ Nevertheless, the benefit of extended rivaroxaban anticoagulation treatment in preventing recurrent VTE has been shown to be consistent regardless of PS score subgroup, as indicated by negative treatment‐by‐subgroup interaction.

Anticoagulation strategies should be determined based not only on the thrombotic risk but also on the bleeding risk. In a post hoc analysis of the Caravaggio study,^18^ an ECOG PS of 2 was an independent risk factor for major bleeding in patients with cancer‐associated VTE, which indicates that patients with cancer‐associated VTE and a high PS score are at a higher risk of major bleeding compared with those with a low PS score. The current study showed that there was no statistical difference with respect to major bleeding between the 18‐month and 6‐month rivaroxaban treatment regardless of PS score. However, extended rivaroxaban treatment resulted in a numerically higher rate of major bleeding rate in the high PS score subgroup. This may be related to the fact that patients with cancer‐associated PE with a high PS score had higher risk of major bleeding than those with a low PS score.^18^ Importantly, there may be some concerns regarding an increased risk of major bleeding with extended anticoagulation therapy among patients with a high PS score.

In general, it is a reasonable option to apply the results of the ONCO PE trial^10^ for patients with a low PS score as well as those with a high PS score in terms of VTE recurrence. On the other hand, considering the importance of identifying suitable patients with cancer‐associated VTE for extended anticoagulation therapy,^22^ and the low statistical power in the current study, clinicians should be cautious for VTE recurrence and bleeding risk from extended anticoagulation therapy in patients with a high PS score, and anticoagulation strategies for these patients should be personalized in individual patients. As such, patients with cancer‐associated acute low‐risk PE should receive extended anticoagulation therapy irrespective of PS score if they do not have a profile of high bleeding risk in clinical practice. In addition, the decision of extended anticoagulation must be individualized, with particular caution exercised in patients with a high PS score whose bleeding risk might compromise the thromboprophylactic benefit.

### Limitations

The current study has several limitations in addition to those already mentioned in the primary report of the ONCO PE trial.^10^ First, the relatively small sample size of the ONCO PE trial may have an impact on the results, especially in the subgroup analysis. Thus, the current analysis should be interpreted as an exploratory approach. Second, based on the previous literature^13^ and the characteristics of the current patient population, we adopted the current threshold of PS score to classify the patients into the low and high PS score subgroups. However, the research results might have been different if classified by other thresholds of PS score. Third, since the current patient population included all patients with cancer‐associated low‐risk PE receiving rivaroxaban in Japan, caution is warranted when extrapolating the current findings to patient populations with different PE severity, anticoagulant therapies, or regional characteristics. Particularly, we should be careful in extrapolating the current findings to patients with more severe PE who potentially could have more limited PS.

## CONCLUSIONS

Extended anticoagulation therapy with rivaroxaban for patients with cancer‐associated low‐risk PE may offer potential benefit in terms of thrombotic risk irrespective of PS score, although there might be some concerns regarding increased risk of major bleeding in patients with a high PS score.

## Sources of Funding

Funding was provided by Bayer Yakuhin, Ltd., which had no role in the study design, data collection, analysis, interpretation, or writing of the report.

## Disclosures

Dr Yamashita received lecture fees from Bayer Yakuhin, Bristol‐Myers Squibb, Pfizer, and Daiichi‐Sankyo, and grant support from Bayer Yakuhin and Daiichi‐Sankyo. Dr Morimoto reports lecturer's fees from Abbott, AstraZeneca, Boehringer Ingelheim, Bristol‐Myers Squibb, Daiichi Sankyo, Japan Lifeline, Pfizer, Tsumura, and UCB; manuscript fees from Pfizer; and is on the advisory board for GlaxoSmithKline, Novartis, and Teijin. Dr Shibata received lecture fees from Novartis Pharmaceuticals KK and Otsuka Pharmaceuticals Co. Ltd. Dr Nishimoto received lecture fees from Bayer Yakuhin, Bristol‐Myers Squibb, Pfizer, and Daiichi‐Sankyo. The remaining authors have no disclosures to report.

## Supporting information

Data S1–S5
Tables S1–S5
Figures S1–S3

## References

[1] Khan F , Tritschler T , Kahn SR , Rodger MA . Venous thromboembolism. Lancet. 2021;398:64–77. doi: 10.1016/S0140-6736(20)32658-1 33984268

[2] Guntupalli SR , Spinosa D , Wethington S , Eskander R , Khorana AA . Prevention of venous thromboembolism in patients with cancer. BMJ. 2023;381:e072715. doi: 10.1136/bmj-2022-072715 37263632

[3] Konstantinides SV , Meyer G , Becattini C , Bueno H , Geersing GJ , Harjola VP , Huisman MV , Humbert M , Jennings CS , Jiménez D , et al. 2019 ESC guidelines for the diagnosis and management of acute pulmonary embolism developed in collaboration with the European Respiratory Society (ERS). Eur Heart J. 2020;41:543–603. doi: 10.1093/eurheartj/ehz405 31504429

[4] Stevens SM , Woller SC , Kreuziger LB , Bounameaux H , Doerschug K , Geersing GJ , Huisman MV , Kearon C , King CS , Knighton AJ , et al. Antithrombotic therapy for VTE disease: second update of the CHEST guideline and expert panel report. Chest. 2021;160:e545–e608. doi: 10.1016/j.chest.2021.07.055 34352278

[5] Lyman GH , Carrier M , Ay C , Di Nisio M , Hicks LK , Khorana AA , Leavitt AD , Lee AYY , Macbeth F , Morgan RL , et al. American Society of Hematology 2021 guidelines for management of venous thromboembolism: prevention and treatment in patients with cancer. Blood Adv. 2021;5:927–974. doi: 10.1182/bloodadvances.2020003442 33570602 PMC7903232

[6] Streiff MB , Holmstrom B , Angelini D , Ashrani A , Buckner T , Diep R , Fertrin KY , Fogerty AE , Crestani NG , Gangaraju R , et al. Cancer‐associated venous thromboembolic disease, version 2.2024, NCCN clinical practice guidelines in oncology. J Natl Compr Cancer Netw. 2024;22:483–506. doi: 10.6004/jnccn.2024.0046 39236759

[7] Farge D , Frere C , Connors JM , Khorana AA , Kakkar A , Ay C , Muñoz A , Brenner B , Prata PH , Brilhante D , et al. 2022 international clinical practice guidelines for the treatment and prophylaxis of venous thromboembolism in patients with cancer, including patients with COVID‐19. Lancet Oncol. 2022;23:e334–e347. doi: 10.1016/S1470-2045(22)00160-7 35772465 PMC9236567

[8] Key NS , Khorana AA , Kuderer NM , Bohlke K , Lee AYY , Arcelus JI , Wong SL , Balaban EP , Flowers CR , Gates LE , et al. Venous thromboembolism prophylaxis and treatment in patients with cancer: ASCO guideline update. J Clin Oncol. 2023;41:3063–3071. doi: 10.1200/jco.23.00294 37075273

[9] Xiong W , Yamashita Y , Horie T , Ono K . The current status and future perspective of extended anticoagulation therapy for cancer‐associated venous thromboembolism. J Thromb Thrombolysis. 2025;58:601–607. doi: 10.1007/s11239-025-03103-4 40299165

[10] Yamashita Y , Morimoto T , Muraoka N , Shioyama W , Chatani R , Shibata T , Nishimoto Y , Ogihara Y , Doi K , Oi M , et al. Rivaroxaban for 18 months versus 6 months in patients with cancer and acute low‐risk pulmonary embolism: an open‐label, multicenter, randomized clinical trial (ONCO PE trial). Circulation. 2025;151:589–600. doi: 10.1161/circulationaha.124.072758 39556015 PMC11875411

[11] Atkinson TM , Andreotti CF , Roberts KE , Saracino RM , Hernandez M , Basch E . The level of association between functional performance status measures and patient‐reported outcomes in cancer patients: a systematic review. Support Care Cancer. 2015;23:3645–3652. doi: 10.1007/s00520-015-2923-2 26314706 PMC4832926

[12] Bersanelli M , Brighenti M , Buti S , Barni S , Petrelli F . Patient performance status and cancer immunotherapy efficacy: a meta‐analysis. Med Oncol. 2018;35:132. doi: 10.1007/s12032-018-1194-4 30128793

[13] Kumar D , Neeman E , Zhu S , Sun H , Kotak D , Liu R . Revisiting the association of ECOG performance status with clinical outcomes in diverse patients with cancer. J Natl Compr Cancer Netw. 2024;22:e237111. doi: 10.6004/jnccn.2023.7111 38653321

[14] Zhang J , Yang L , Tian H , Xu R , Liu D . The value of performance status in predicting venous thromboembolism in lung cancer patients treated with immune checkpoint inhibitors. Eur J Oncol Nurs. 2024;69:102527. doi: 10.1016/j.ejon.2024.102527 38377652

[15] Guven DC , Aksun MS , Sahin TK , Aktepe OH , Yildirim HC , Taban H , Ceylan F , Kertmen N , Arik Z , Dizdar O , et al. Poorer baseline performance status is associated with increased thromboembolism risk in metastatic cancer patients treated with immunotherapy. Support Care Cancer. 2021;29:5417–5423. doi: 10.1007/s00520-021-06139-3 33709186

[16] Farmakis IT , Barco S , Mavromanoli AC , Konstantinides SV , Valerio L . Performance status and long‐term outcomes in cancer‐associated pulmonary embolism: insights from the Hokusai‐VTE cancer study. JACC CardioOncol. 2022;4:507–518. doi: 10.1016/j.jaccao.2022.07.008 36444229 PMC9700256

[17] Sallah S , Husain A , Sigounas V , Wan J , Turturro F , Sigounas G , Nguyen NP . Plasma coagulation markers in patients with solid tumors and venous thromboembolic disease receiving oral anticoagulation therapy. Clin Cancer Res. 2004;10:7238–7243. doi: 10.1158/1078-0432.CCR-04-0445 15534097

[18] Vedovati MC , Giustozzi M , Munoz A , Bertoletti L , Cohen AT , Klok FA , Connors JM , Bauersachs R , Brenner B , Campanini M , et al. Risk factors for recurrence and major bleeding in patients with cancer‐associated venous thromboembolism. Eur J Intern Med. 2023;112:29–36. doi: 10.1016/j.ejim.2023.02.003 36774305

[19] Hopewell S , Chan AW , Collins GS , Hróbjartsson A , Moher D , Schulz KF , Tunn R , Aggarwal R , Berkwits M , Berlin JA , et al. CONSORT 2025 statement: updated guideline for reporting randomized trials. Jama. 2025;333:1998–2005. 40228499 10.1001/jama.2025.4347

[20] Raskob GE , van Es N , Verhamme P , Carrier M , Di Nisio M , Garcia D , Grosso MA , Kakkar AK , Kovacs MJ , Mercuri MF , et al. Edoxaban for the treatment of cancer‐associated venous thromboembolism. N Engl J Med. 2018;378:615–624. doi: 10.1056/NEJMoa1711948 29231094

[21] Schulman S , Kearon C ; Subcommittee on Control of Anticoagulation of the Scientific and Standardization Committee of the International Society on Thrombosis and Haemostasis . Definition of major bleeding in clinical investigations of antihemostatic medicinal products in non‐surgical patients. J Thromb Haemost. 2005;3:692–694. doi: 10.1111/j.1538-7836.2005.01204.x 15842354

[22] Xiong W , Yamashita Y , Morimoto T , Muraoka N , Umetsu M , Nishimoto Y , Takada T , Ogihara Y , Nishikawa T , Ikeda N , et al. Utility of the modified Ottawa score for identification of more preferable candidates of extended anticoagulation therapy in cancer‐associated isolated distal deep vein thrombosis: insight from the ONCO DVT study. J Thromb Haemost. 2024;22:3542–3551. doi: 10.1016/j.jtha.2024.09.003 39284385

