# Supplementary material for: Extended Anticoagulation Therapy With Rivaroxaban for Cancer‐Associated Low‐Risk Pulmonary Embolism According to Different Performance Status Scores: Insights From the ONCO PE Randomized Trial
Source: J Am Heart Assoc. 2026 Feb 27;15(6):e045541. doi: 10.1161/JAHA.125.045541 (PMC13055693; doi:10.1161/JAHA.125.045541)
Supplement: Supplementary file 1 — Data S1–S5 Tables S1–S5 Figures S1–S3 [file JAH3-15-e045541-s001.zip › data supplement for publication.pdf]

SUPPLEMENTAL MATERIAL

Supplemental Methods

Data S1: Study Organization

Principal Investigators: Yugo YAMASHITA, Department of Cardiovascular Medicine, Graduate School of Medicine and Faculty of Medicine Kyoto University; Takeshi KIMURA (Co-Principal Investigators), Department of Cardiology, Hirakata Kohsai Hospital

Protocol Committee: Yugo YAMASHITA, Department of Cardiovascular Medicine, Graduate School of Medicine and Faculty of Medicine Kyoto University: Takeshi KIMURA (Co-Principal Investigators), Department of Cardiology, Hirakata Kohsai Hospital; Takeshi MORIMOTO, Department of Clinical Epidemiology, Hyogo College of Medicine

Steering Committee: Nao MURAOKA, Division of Cardiology, Shizuoka Cancer Center; Wataru SHIOYAMA, Department of Cardiovascular Medicine, Shiga University of Medical Science; Ryuki CHATANI, Department of Cardiovascular Medicine, Kurashiki Central Hospital; Tatsuhiro SHIBATA, Division of Cardiovascular Medicine, Department of Internal Medicine, Kurume University School of Medicine; Yuji NISHIMOTO, Division of Cardiology, Osaka General Medical Center

Clinical Events Committee: Yuji NISHIMOTO, Division of Cardiology, Osaka General Medical Center; Maki OI, Department of Cardiology, Japanese Red Cross Otsu Hospital; Reo HATA, Department of Cardiovascular Medicine, Graduate School of Medicine and Faculty of Medicine Kyoto University; Kitae KIM, Department of Cardiovascular Medicine, Kobe City Medical Center General Hospital

Data Safety Monitoring Committee: Yasuhiro HAMATANI, Department of Cardiovascular Medicine, NHO Kyoto Medical Center; Hidenori YAKU, Department of Cardiovascular Medicine, Mitsubishi Kyoto Hospital

Clinical Research Organization: MID, Inc. and Department of Cardiovascular Medicine, Graduate School of Medicine and Faculty of Medicine Kyoto University

Research Operations Staff: Nozomi Tanaka, Department of Cardiovascular Medicine, Graduate School of Medicine and Faculty of Medicine Kyoto University

Monitoring officers: Yukiko NAKANO, Department of Cardiovascular Medicine, Graduate School of Medicine and Faculty of Medicine Kyoto University

Auditors: Chikashi TAKEDA, Department of Anesthesia, Kyoto University Hospital and Department of Pharmacoepidemiology, Kyoto University Graduate School of Medicine and Public Health

Principal Statistician: Takeshi MORIMOTO, Department of Clinical Epidemiology, Hyogo College of Medicine

Data S2: Participating Centers

Department of Cardiovascular Medicine, Kyoto University Hospital (Yugo YAMASHITA), Department of Cardiovascular Medicine, Shizuoka Cancer Center (Nao MURAOKA), Department of Cardiovascular Medicine, Shiga University of Medical Science (Wataru SHIOYAMA), Department of Cardiovascular Medicine, Kurashiki Central Hospital (Kazushige KADOTA), Division of Cardiovascular Medicine, Department of Internal Medicine, Kurume University Hospital (Yoshihiro FUKUMOTO), Department of Cardiovascular Medicine, Mie University Hospital (Yoshito OGIHARA), Division of Cardiology, Osaka General Medical Center (Yuji NISHIMOTO), Department of Cardiology, NHO Kyoto Medical Center (Masaharu AKAO), Department of Cardiology, Japanese Red Cross Otsu Hospital (Kazuaki KAITANI), Department of Onco-Cardiology/Cardiovascular Medicine, Cancer Institute Hospital of Japanese Foundation for Cancer Research (Taro SHIGA), Department of Cardiovascular Medicine, Kumamoto University Hospital (Kenichi TSUJITA), Department of Cardiovascular Medicine, Kobe City Medical Center General Hospital (Yutaka FURUKAWA), Department of Cardiology, St. Marianna University School of Medicine (Yasuhiro TANABE), Department of Cardiovascular Medicine, Gunma University (Norimichi KOITABASHI), Department of Cardiovascular Medicine, Tokyo Women's Medical University Hospital (Atsushi SUZUKI), Department of Cardiovascular Medicine, Nagasaki University Hospital (Koji MAEMURA), Department of Cardiovascular Medicine, Nara Medical University (Yoshihiko SAITO), Department of Cardiovascular Medicine, Fujisawa City Hospital (Kengo TSUKAHARA), Department of Cardiovascular Medicine, National Cancer Center Hospital (Masaaki SHOJI), Department of Cardiovascular Medicine,Tenri Hospital (Toshihiro TAMURA), Division of Cardiovascular Medicine, Toho University Omori Medical Center (Shinji HISATAKE), Department of Cardiovascular Medicine, Hyogo Prefectural Amagasaki General Medical Center (Yukihiro SATO), Department of Cardiology, Yokohama City University, Yokohama (Tomoaki ISHIGAMI), Department of Onco-Cardiology, Osaka International Cancer Institute (Masafumi FUJITA), Department of Cardiovascular Medicine, University Hospital Kyoto Prefectural University of Medicine (Satoaki MATOBA), Department of Cardiovascular Medicine, Kohka Public Hospital (Tomohiro DOUKA), Department of Cardiovascular Medicine, Kokura Memorial Hospital (Kenji ANDO), Department of Cardiology, Shizuoka City Shizuoka Hospital (Ryuzo

NAWADA), Department of Cardiovascular Medicine, Mitsubishi Kyoto Hospital (Shinji MIKI), Department of Cardiovascular Medicine, Shiga General Hospital (Tsuyoshi INOUE), Department of Cardiovascular Medicine, Saku Central Hospital Advanced Care Center (Yoshikazu YAZAKI), Shimada General Medical Center (Takeshi AOYAMA)

Data S3: Detailed Inclusion Criteria and Exclusion Criteria

Inclusion criteria

Patients with active cancer who were newly diagnosed with pulmonary embolism (PE), were scheduled for treatment with anticoagulation therapy, and satisfied all the following inclusion criteria:

1. Male or female patients aged 20-80 years old
2. Patients who were newly diagnosed with PE confirmed by contrast-enhanced computed tomography
3. Patients with active cancer* at randomization.
4. Patients with a simplified version of the Pulmonary Embolism Severity Index (PESI) score of 1.
5. Patients who had provided informed consent.

Note: The simplified PESI score was calculated based on age greater than 80 years, history of cancer, history of chronic cardiopulmonary disease, heart rate of 110 beats/minute or greater, systolic blood pressure less than 100 mmHg, and arterial oxygen saturation less than 90% at the time of diagnosis.

Note: Cancer in this study included all malignant tumors, including epithelial carcinomas, sarcomas, and hematologic malignancies such as leukemia.

Note: Active cancer satisfied one of the following criteria:

1. Newly diagnosed with cancer within 6 months of randomization
2. Cancer treatment (surgery, chemotherapy, radiotherapy, etc.) performed within 6 months of randomization
3. Currently receiving cancer treatment (surgery, chemotherapy, radiotherapy, etc.)
4. Has a recurrence, local invasion, or distant metastases
5. Patients with ahematopoietic malignancy who have not achieved complete remission

Exclusion criteria

Patients who had any of the following criteria are excluded:

1. Contraindicated patients for rivaroxaban (clinically significant liver disease, bacterial endocarditis, active bleeding, inadequate contraceptive measures if of childbearing potential, concomitant use of strong cytochrome P-450 3A4 inhibitors or inducers or P-glycoprotein inhibitors or inducers, etc.)
2. Patients currently on oral anticoagulation therapy (warfarin, direct oral anticoagulant [DOAC]) for treatment/prevention of a recurrence of venous thromboembolism (VTE) for purposes other than the index event; or treated with oral anticoagulation therapy (warfarin, DOAC) for indications other than a VTE at the time of the diagnosis.
3. Patients who were expected to have a life prognosis of 6 months or less.
4. Patients who were regarded as not appropriate for participation in the study by the attending physician.

Data S4: Definition of the baseline characteristics

Place of onset was classified as out-of-hospital onset, in-hospital onset after surgery, and in-hospital onset other than after surgery. Patients admitted to the hospital with any symptoms, who could not be diagnosed with PE at admission but were diagnosed with PE after admission, and it was clinically assumed that the symptoms that caused the PE were present at admission, were classified as an out-of-hospital onset. However, if the onset of the PE at admission could not be clinically assumed, and the patients were assumed to have developed the PE after admission, they were classified as an in-hospital onset other than after surgery. An in-hospital onset after surgery was classified when the patients had a history of surgery 2 months or less before the diagnosis of the PE and were continuously hospitalized since the surgery. All other in-hospital onset cases were classified as an in-hospital onset other than that after surgery. Home treatment was defined as immediate discharge directly from the emergency department or the outpatient clinic within 12 hours after the first visit to the hospital among patients who were diagnosed with PE out of hospital. All other management was defined as in-hospital treatment including treatment in hospital among patients who were diagnosed with PE in hospital. Right ventricular (RV) dysfunction was defined as the presence of an enlarged RV finding (right ventricular /left ventricular ≥0.9) on CT, or the existence of an estimated systolic pulmonary arterial pressure of ≥40 mmHg by echocardiography. Hypertension was diagnosed if the peripheral blood pressure was >140/90 mmHg or if the patient was taking medication for hypertension. Diabetes was diagnosed based on a blood glucose level ≥200 mg/dl 2 or more hours after loading in a glucose tolerance test, casual blood glucose ≥200 mg/dl, fasting blood glucose ≥126 mg/dl, hemoglobin A1c ≥6.5%, or if the patient has already been clinically diagnosed with diabetes or was taking medication to treat diabetes. Dyslipidemia was diagnosed based on a total cholesterol level ≥240 mg/dl, high-density lipoprotein cholesterol level <40 mg/dl, or taking statins. A history of major bleeding was diagnosed according to the definition of the International Society on Thrombosis and Haemostasis (ISTH) criteria, which consisted of fatal bleeding, symptomatic bleeding in a critical area or organ, and bleeding causing a reduction in the hemoglobin level by at least 2 g/dL or leading to a transfusion of at least 2 units of whole blood or red cells. Anemia was defined as a hemoglobin level <13 g/dL for men and <12 g/dL for women according to the standard World Health Organization classification of anemia. Antiplatelet drugs included aspirin, ticlopidine, clopidogrel, prasugrel, ticagrelor, and cilostazol.

Data S5: Secondary Endpoints and Definition of the Endpoints

■ Major secondary endpoint

- Major bleeding event at 18 months after the diagnosis

■ Other secondary endpoints

These endpoints are evaluated 18 months after diagnosis.

✓ Deaths from all causes

✓ Symptomatic recurrent VTE events

✓ All clinically relevant bleeding events (major and non-major bleeding events)

● **Death**

Cause of death was classified into the following 5 categories. PE-related deaths, deaths due to cancer, cardiovascular deaths, deaths due to bleeding, and deaths due to other known causes.

✓ PE-related death: Death due to PE diagnosed at autopsy, death followed a clinically severe PE, or death unexplained by other than PE

✓ Cancer: Deaths conceivably directly associated with cancer (example: brain herniation, coma, brain tumor causing respiratory arrest), and debilitating (gradual) death due to the progression of cancer

✓ Cardiovascular death: All cardiac and vascular deaths (myocardial infarction, low-output heart failure, lethal arrhythmia, cerebrovascular disease, ruptured aortic aneurysm, dissecting aneurysm, etc.)

✓ Bleeding: Deaths directly caused by hemorrhage (for example: cerebral hemorrhage causing brain herniation, gastrointestinal hemorrhage resulting in death due to hemorrhagic shock, etc.)

✓ Other known causes: Deaths due to known causes not listed above. These include infection, renal failure, lung disease, intraoperative death, accident, suicide, trauma, etc.

● **Symptomatic recurrent VTE**

New or newly worsening PE or DVT symptoms, and new thrombi found on imaging tests (ultrasonography of lower limb vein system, computed tomography examination, pulmonary perfusion scintigraphy, pulmonary angiography, venography), or thrombi that have worsened over time compared to the most recent image were defined as a symptomatic VTE recurrence. Symptomatic VTE recurrence was not determined solely on the appearance or worsening of thrombus images on imaging without new or worsening symptoms. Deep vein thrombosis other than that in the extremities was included as symptomatic recurrent VTE if the patients showed any symptoms related to thrombosis.

● **Bleeding/Hemorrhagic Events**

The severity of bleeding was classified using the International Society on Thrombosis and Haemostasis (ISTH) bleeding classification

Major bleeding: Symptoms indicating bleeding, and applicable to any of the following:

✓ Fatal bleeding

- ✓ Symptomatic bleeding in a critical area or organ (intracranial, intraspinal, intraocular, retroperitoneal, intraarticular or pericardial, or intramuscular with compartment syndrome)
- ✓ Bleeding causing a fall in the hemoglobin level of 2 g/dL or more or leading to a transfusion of two or more units of whole blood or red cells.

All clinically relevant bleeding events

All clinically relevant bleeding events included major and clinically relevant non-major bleeding events.

Clinically relevant non-major bleeding: Clinically relevant non-major bleeding was defined as a clinically overt bleed (including bleeds detected only with imaging diagnostics) that did not meet the criteria for a major bleed but led to at least one of the following:

- ✓ A physician-guided medical intervention
- ✓ A hospital admission or further treatment for bleeding
- ✓ Face-to-face medical examination by physician (not simply telephone or electronic communication)

**Table S1. Detailed cancer types in the low and high PS score subgroups**		
**Cancer types, No. (%)**	**Low PS score (PS=0) (N=79)**	**High PS score (PS≥1) (N=99)**
**Lung**	7 (8.9)	9 (9.1)
**Colon**	11 (13.9)	11 (11.1)
**Stomach**	3 (3.8)	7 (7.1)
**Uterus**	8 (10.1)	13 (13.1)
**Blood**	1 (1.3)	3 (3.0)
**Ovary**	10 (12.7)	9 (9.1)
**Prostate**	0 (0.0)	3 (3.0)
**Breast**	6 (7.6)	5 (5.1)
**Pancreas**	6 (7.6)	13 (13.1)
**Bladder**	4 (5.1)	2 (2.0)
**Kidney/ureter**	3 (3.8)	4 (4.0)
**Esophagus**	3 (3.8)	2 (2.0)
**Gall bladder/bile duct**	3 (3.8)	5 (5.1)
**Skin**	2 (2.5)	0 (0.0)
**Liver**	3 (3.8)	1 (1.0)
**Multiple**	2 (2.5)	3 (3.0)
**Others**	7 (8.9)	9 (9.1)

Abbreviation: PS: performance status.

Table S2. Baseline clinical characteristics comparing 18-month and 6-month rivaroxaban groups in the low and high PS score subgroups						
	Low PS score (PS=0) (N=79)			High PS score (PS≥1) (N=99)		
	18-month rivaroxaban (N=37)	6-month rivaroxaban (N=42)	P value	18-month rivaroxaban (N=52)	6-month rivaroxaban (N=47)	P value
**Demographics**						
Age, years	65.4 ± 11.0	64.6 ± 10.4	0.77	66.9 ± 10.0	65.7 ± 10.7	0.57
Age ≥75 years, n (%)	8 (21.6)	8 (19.0)	0.78	15 (28.8)	9 (19.1)	0.26
Men, n (%)	16 (43.2)	20 (47.6)	0.70	26 (50.0)	21 (44.7)	0.60
Body weight, kg	60.8 ± 12.1	61.2 ± 12.1	0.88	60.3 ± 10.7	58.4 ± 11.1	0.37
Body weight ≤60 kg, n (%)	19 (51.4)	22 (52.4)	0.93	25 (48.1)	30 (63.8)	0.12
Body mass index, kg/m^2	23.0 ±2.9	23.1 ± 3.6	0.88	23.0 ± 4.8	22.9 ± 3.4	0.87
**Cancer status, n (%)**						
Newly diagnosed cancer within 6 months	22 (59.5)	22 (52.4)	0.53	35 (67.3)	23 (48.9)	0.06
Surgery within 6 months	17 (45.9)	17 (40.5)	0.62	20 (38.5)	15 (31.9)	0.50
Chemotherapy within 6 months	26 (70.3)	24 (57.1)	0.23	30 (57.7)	27 (57.4)	0.98
Radiotherapy within 6 months	1 (2.7)	4 (9.5)	0.21	2 (3.8)	6 (12.8)	0.10
Recurrent cancer	2 (5.4)	8 (19.0)	0.07	4 (7.7)	13 (27.7)	0.01
Metastasis	8 (21.6)	9 (21.4)	0.98	26 (50.0)	24 (51.1)	0.92
**Comorbidities, n (%)**						
Hypertension	12 (32.4)	16 (38.1)	0.60	18 (34.6)	21 (44.7)	0.31
Diabetes	5 (13.5)	2 (4.8)	0.17	10 (19.2)	10 (21.3)	0.80
Dyslipidemia	6 (16.2)	9 (21.4)	0.56	12 (23.1)	11 (23.4)	0.97
History of stroke	0 (0.0)	0 (0.0)	-	1 (1.9)	0 (0.0)	0.34
History of venous thromboembolism	1 (2.7)	2 (4.8)	0.63	1 (1.9)	3 (6.4)	0.26
Autoimmune disorder	3 (8.1)	3 (7.1)	0.87	3 (5.8)	3 (6.4)	0.90

Varicose veins of lower extremities	1 (2.7)	0 (0.0)	0.28	0 (0.0)	0 (0.0)	-
History of major bleeding	2 (5.4)	2 (4.8)	0.90	5 (9.6)	1 (2.1)	0.12
Chronic kidney disease	3 (8.1)	4 (9.5)	0.83	1 (1.9)	4 (8.5)	0.14
**Predisposing factors, n (%)**						
Transient factors	6 (16.2)	14 (33.3)	0.08	13 (25.0)	9 (19.1)	0.48
Immobilization	1 (2.7)	4 (9.5)	0.21	3 (5.8)	4 (8.5)	0.60
Recent surgery within 2 months	4 (10.8)	9 (21.4)	0.20	10 (19.2)	6 (12.8)	0.38
Central venous catheterization	0 (0.0)	0 (0.0)	-	0 (0.0)	1 (2.1)	0.29
**Settings of onset, n (%)**						
Out-of-hospital onset	29 (78.4)	24 (57.1)	0.05	23 (44.2)	21 (44.7)	0.96
Home treatment	21 (56.8)	19 (45.2)	0.31	15 (28.8)	11 (23.4)	0.54
In-hospital onset	8 (21.6)	18 (42.9)	0.05	29 (55.8)	26 (55.3)	0.96
In-hospital onset after surgery	3 (8.1)	7 (16.7)	0.25	10 (19.2)	9 (19.1)	0.99
**Presentation at diagnosis**						
Systolic blood pressure, mm Hg	132.6 ± 17.1	128.5 ± 18.1	0.31	124.0 ± 16.6	122.6 ± 14.5	0.66
Heart rate, bpm	78.4 ± 12.4	80.7 ± 12.0	0.39	82.4 ± 11.2	82.4 ± 13.1	0.99
Oxygen saturation, %	97.7 ± 1.2	97.4 ± 1.3	0.27	96.7 ± 1.7	97.3 ± 1.3	0.06
Symptomatic, n (%)	4 (10.8)	3 (7.1)	0.57	9 (17.3)	6 (12.8)	0.53
**PE characteristics, n (%)**						
Thrombus location						
Central	3 (8.1)	2 (4.8)	0.54	5 (9.6)	4 (8.5)	0.85
Main	4 (10.8)	4 (9.5)	0.85	8 (15.4)	6 (12.8)	0.70
Lobar	12 (32.4)	15 (35.7)	0.76	15 (28.8)	12 (25.5)	0.71
Segmental	8 (21.6)	16 (38.1)	0.11	17 (32.7)	12 (28.6)	0.67
Subsegmental	10 (27.0)	5 (11.9)	0.09	7 (13.5)	13 (27.7)	0.08
RV dysfunction	3 (8.1)	1 (2.4)	0.25	8 (15.4)	3 (6.4)	0.16
RV/LV≥0.9	3 (8.1)	1 (2.4)	0.25	5 (9.6)	3 (6.4)	0.56

Concomitant DVT	23 (62.2)	22 (52.4)	0.38	27 (51.9)	32 (68.1)	0.10
**Laboratory tests at diagnosis**						
Hemoglobin, g/dL	11.3 ± 2.4	12.2 ± 1.8	0.06	11.2 ± 1.5	11.0 ± 2.2	0.60
Anemia, n. (%)	25 (67.6)	20 (47.6)	0.07	40 (76.9)	33 (70.2)	0.45
Platelet count, ×100 000/μL	20.3 ± 9.8	23.8 ± 9.0	0.10	24.6 ± 9.6	23.5 ± 13.8	0.64
Platelet count <100 000/μL, n (%)	3 (8.1)	0 (0.0)	0.06	2 (3.8)	6 (12.8)	0.10
Creatinine clearance, mL/min	87.7 ± 29.2	83.9 ± 28.5	0.56	80.6 (61.2-101.1)	78.5 (59.6-99.0)	0.75
Creatinine clearance ≤50 mL/min, n (%)	4 (10.8)	2 (4.8)	0.31	2 (3.8)	3 (6.4)	0.57
D-dimer (n=166), μg/mL	6.0 (3.5-11.1)	5.2 (2.6-10.9)	0.63	10.3 (5.1-22.1)	9.9 (5.5-18.6)	0.57
NT-proBNP (n=48), pg/mL	102.0 (66.0-240.4)	65.0 (36.3-138.0)	0.19	196.0 (57.8-278.0)	86.2 (61.0-125.0)	0.16
Troponin I (n=53), ng/dL	0.010 (0.005-2.510)	0.955 (0.010-10.00)	0.33	0.010 (0.005-10.00)	0.022 (0.010-8.750)	0.83
**Concomitant medication, n (%)**						
Antiplatelet	2 (5.4)	0 (0.0)	0.13	1 (1.9)	2 (4.3)	0.50
Statins	2 (5.4)	5 (11.9)	0.31	10 (19.2)	4 (8.5)	0.13
Steroid	5 (13.5)	3 (7.1)	0.35	6 (11.5)	10 (21.3)	0.19
NSAIDs	4 (10.8)	4 (9.5)	0.85	10 (19.2)	7 (14.9)	0.57
Proton pump inhibitor	16 (43.2)	8 (19.0)	0.02	30 (57.7)	27 (57.4)	0.98

Continuous variables were compared using Student t-test or Mann-Whitney U test, based on whether or not their distributions were normal. The comparison of categorical variables was conducted using the $\chi 2$ test or Fisher exact test.

Abbreviations: PS: performance status; RV: right ventricular; LV: left ventricular; DVT: deep vein thrombosis; NT-proBNP: N-terminal-pro B-type natriuretic peptide; NSAIDs: non-steroidal anti-inflammatory drugs.

**Table S3. Detailed sites of major bleeding in the low and high PS score subgroups**				
**Sites of major bleeding, n (%)**	**Major bleeding in the low PS score subgroup (N=4)**		**Major bleeding in the high PS score subgroup (N=8)**	
	18-month rivaroxaban (N=1)	6-month rivaroxaban (N=3)	18-month rivaroxaban (N=6)	6-month rivaroxaban (N=2)
**Upper gastrointestinal**	0 (0.0)	0 (0.0)	1 (16.7)	1 (50.0)
**Lower gastrointestinal**	0 (0.0)	2 (66.7)	2 (33.3)	0 (0.0)
**Body cavity**	1 (100.0)	0 (0.0)	2 (33.3)	1 (50.0)
**Genitals**	0 (0.0)	1 (33.3)	1 (16.7)	0 (0.0)

Abbreviations: PS: performance status.

Table S4. Clinical outcomes for other secondary endpoints						
	N of patients with event		Difference in event rates (95% CI)	Odds ratio (95% CI)	P value	$P_{interaction}$
	18-month rivaroxaban (N=89)	6-month rivaroxaban (N=89)				
**All-cause death, No. (%)**						
Low PS score (PS=0)	9/37 (24.3%)	5/42 (11.9%)	12.4% (-5.0%– 29.1%)	2.38 (0.72-7.88)	0.16	0.24
High PS score (PS≥1)	20/52 (38.5%)	18/47 (38.3%)	0.2% (-18.7%–19.0%)	1.01 (0.45-2.27)	0.99	
**Symptomatic recurrent VTE, No. (%)**						
Low PS score (PS=0)	0/37 (0.0%)	3/42 (7.1%)	-7.1% (-16.4%–3.3%)	-	0.99	0.99
High PS score (PS≥1)	1/52 (1.9%)	1/47 (2.1%)	-0.2% (-7.9%–7.1%)	0.90 (0.06-14.84)	0.94	
**All clinically relevant bleeding, No. (%)**						
Low PS score (PS=0)	8/37 (21.6%)	5/42 (11.9%)	9.7% (-7.2%–26.1%)	2.04 (0.60-6.90)	0.25	0.22
High PS score (PS≥1)	12/52 (23.1%)	13/47 (27.7%)	-4.6 %(-21.5%–12.5%)	0.79 (0.32-1.95)	0.60	

Abbreviations: PS: performance status; CI: confidence interval; VTE: venous thromboembolism.

Table S5. Clinical outcomes in all spectrum of PS classification						
	N of patients with event		Difference in the event rates (95% CI)	Odds ratio (95% CI)	P value	$P_{interaction}$
	18-month rivaroxaban (N=89)	6-month rivaroxaban (N=89)				
**Primary endpoint (recurrent VTE)**						
PS=0	1/37 (2.7%)	8/42 (19.0%)	-16.3% (-29.1%— -1.5%)	0.12 (0.01-0.99)	0.049	0.82
PS=1	1/42 (2.4%)	7/40 (17.5%)	-15.1% (-27.9%—-1.1%)	0.12 (0.01-0.98)	0.048	
PS=2	2/6 (33.3%)	2/4 (50.0%)	-16.7% (-64.7%–39.7%)	0.50 (0.04-6.68)	0.60	
PS=3	1/4 (25.0%)	0/3 (0.0%)	25.0% (-38.2%–64.8%)	-	-	
**Major secondary endpoint (major bleeding)**						
PS=0	1/37 (2.7%)	3/42 (7.1%)	-4.4% (-14.9%— 7.0%)	0.36 (0.04-3.63)	0.39	0.84
PS=1	3/42 (7.1%)	2/40 (5.0%)	2.1% (-9.6—13.5%)	1.46 (0.23-9.24)	0.69	
PS=2	1/6 (16.7%)	0/4 (0.0%)	16.7% (-34.0%–50.6%)	-	-	
PS=3	2/4 (50.0%)	0/3 (0.0%)	50.0% (-23.2%–83.2%)	-	-	

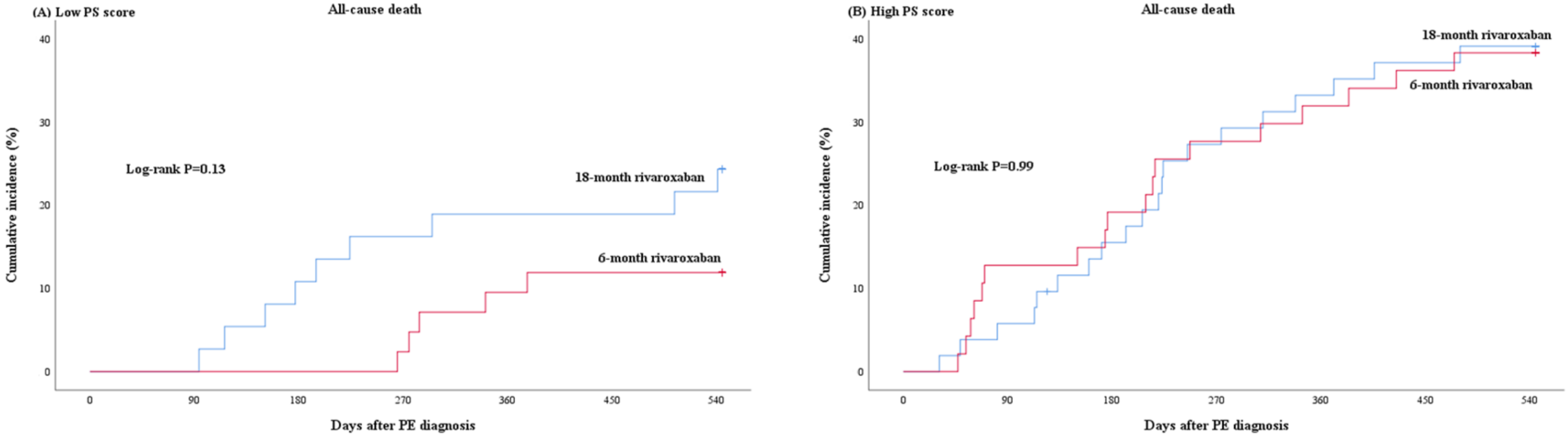

	0 day	180 days	360 days	545 days
**18-month rivaroxaban**				
N of patients with event		4	7	9
N of patients at risk	37	33	30	27
Cumulative incidence		10.8%	18.9%	24.3%
**6-month rivaroxaban**				
N of patients with event		0	4	5
N of patients at risk	42	42	38	36
Cumulative incidence		0.0%	9.5%	11.9%

	0 day	180 days	360 days	545 days
**18-month rivaroxaban**				
N of patients with event		8	17	20
N of patients at risk	52	43	34	30
Cumulative incidence		15.5%	33.2%	39.1%
**6-month rivaroxaban**				
N of patients with event		9	15	18
N of patients at risk	47	38	32	28
Cumulative incidence		19.1%	31.9%	38.3%

Figure S1. Kaplan-Meier curves for all-cause death compared between 18-month and 6-month rivaroxaban treatment groups stratified by the PS score.

Figure S1(A) : Low PS score subgroup; Figure S1(B): High PS score subgroup. Abbreviation: PS: performance status; PE: pulmonary embolism.

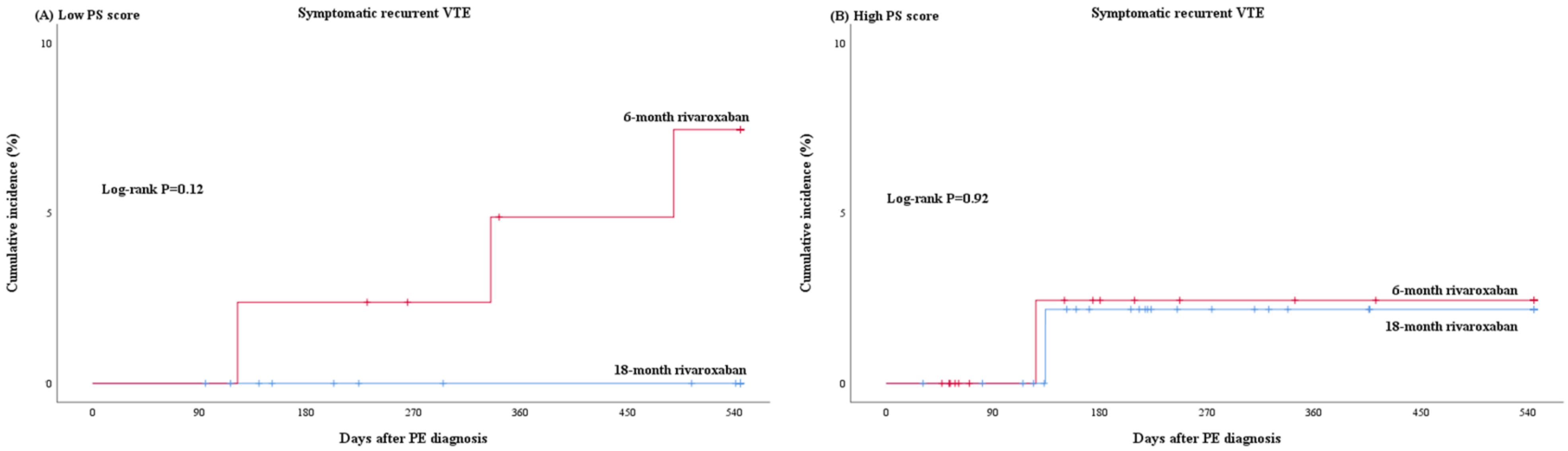

	0 day	180 days	360 days	545 days
**18-month rivaroxaban**				
N of patients with event		0	0	0
N of patients at risk	37	33	30	27
Cumulative incidence		0.0%	0.0%	0.0%
**6-month rivaroxaban**				
N of patients with event		1	2	3
N of patients at risk	42	41	37	35
Cumulative incidence		2.4%	4.9%	7.5%

	0 day	180 days	360 days	545 days
**18-month rivaroxaban**				
N of patients with event		1	1	1
N of patients at risk	52	42	32	29
Cumulative incidence		2.2%	2.2%	2.2%
**6-month rivaroxaban**				
N of patients with event		1	1	1
N of patients at risk	47	37	34	32
Cumulative incidence		2.4%	2.4%	2.4%

Figure S2. Kaplan-Meier curves for symptomatic recurrent VTE compared between 18-month and 6-month rivaroxaban treatment groups stratified by the PS score.

Figure S2(A) : Low PS score subgroup; Figure S2(B): High PS score subgroup. Abbreviation: PS: performance status; VTE: venous thromboembolism; PE: pulmonary embolism

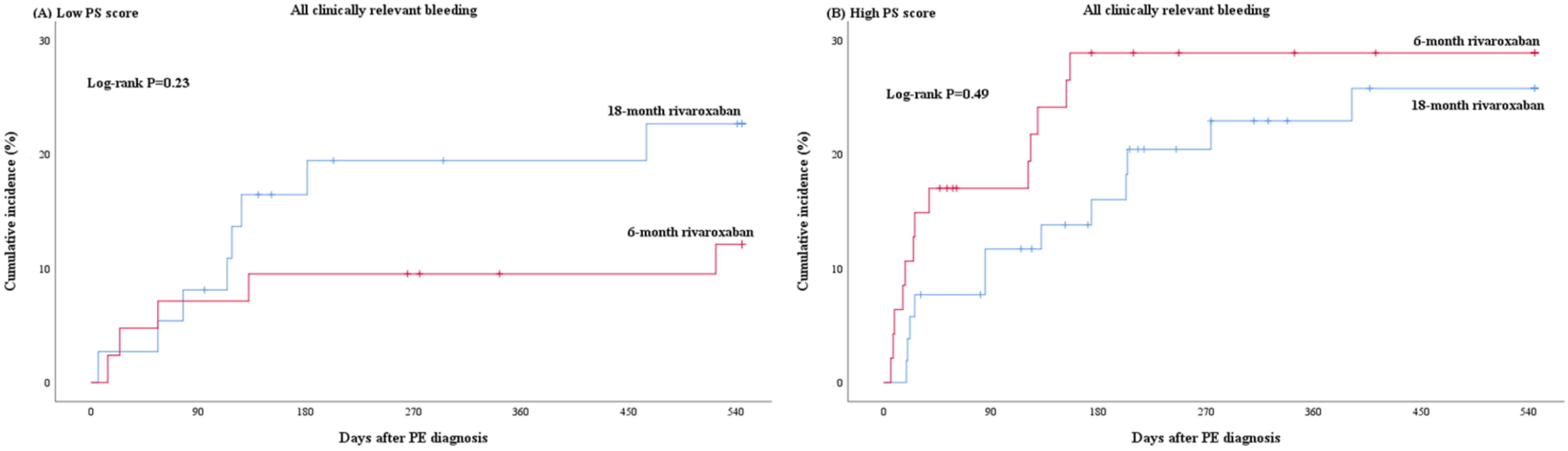

	0 day	180 days	360 days	545 days
**18-month rivaroxaban**				
N of patients with event		6	7	8
N of patients at risk	37	28	25	22
Cumulative incidence		16.5%	19.4%	22.7%
**6-month rivaroxaban**				
N of patients with event		4	4	5
N of patients at risk	42	38	35	33
Cumulative incidence		9.5%	9.5%	12.1%

	0 day	180 days	360 days	545 days
**18-month rivaroxaban**				
N of patients with event		8	11	12
N of patients at risk	52	38	27	24
Cumulative incidence		16.0%	22.9%	25.8%
**6-month rivaroxaban**				
N of patients with event		13	13	13
N of patients at risk	47	29	26	24
Cumulative incidence		28.9%	28.9%	28.9%

Figure S3. Kaplan-Meier curves for all clinically relevant bleeding compared between 18-month and 6-month rivaroxaban treatment groups stratified by the PS score.

Figure S3(A) : Low PS score subgroup; Figure S3(B): High PS score subgroup. Abbreviation: PS: performance status; PE: pulmonary embolism
